# Health layering of self-help groups: impacts on reproductive, maternal, newborn and child health and nutrition in Bihar, India

**DOI:** 10.7189/jogh.10.021007

**Published:** 2020-12

**Authors:** Kala M Mehta, Laili Irani, Indrajit Chaudhuri, Tanmay Mahapatra, Janine Schooley, Sridhar Srikantiah, Safa Abdalla, Victoria Ward, Suzan L Carmichael, Jason Bentley, Andreea Creanga, Jess Wilhelm, Usha Kiran Tarigopula, Debarshi Bhattacharya, Yamini Atmavilas, Priya Nanda, Yingjie Weng, Kevin T Pepper, Gary L Darmstadt, for the Ananya Study Group, Yamini Atmavilas, Debarshi Bhattacharya, Jason Bentley, Evan Borkum, Suzan Carmichael, Indrajit Chaudhuri, Andreea Creanga, Gary L. Darmstadt, Priyanka Dutt, Laili Irani, Tanmay Mahapatra, Kala M. Mehta, Radharani Mitra, Wolfgang A. Munar, Priya Nanda, Kevin T. Pepper, Hina Raheel, Anu Rangarajan, Niranjan Saggurti, Padmapriya Sastry, Hemant Shah, Sridhar Srikantiah, Usha Kiran Tarigopula, Victoria Ward, Yingjie Weng, Dilys Walker, Jess Wilhelm

**Affiliations:** 1 Department of Pediatrics, Stanford University School of Medicine, Stanford, California, USA; 2 Department of Epidemiology and Biostatistics, University of California San Francisco, San Francisco, California, USA; 3 Population Council, New Delhi, India; 4 Project Concern International, Delhi, India and San Diego, California, USA; 5 CARE India, Patna, India; 6 Center for Population Health Sciences, Stanford University School of Medicine, Palo Alto, California, USA; 7 Quantitative Sciences Unit, Department of Medicine, Stanford University School of Medicine, Stanford, California, USA; 8 Department of International Health, Johns Hopkins Bloomberg School of Public Health, Baltimore, Maryland, USA; 9 Bill and Melinda Gates Foundation, Delhi, India

## Abstract

**Background:**

Self-help group (SHG) interventions have been widely studied in low and middle income countries. However, there is little data on specific impacts of health layering, or adding health education modules upon existing SHGs which were formed primarily for economic empowerment. We examined three SHG interventions from 2012-2017 in Bihar, India to test the hypothesis that health-layering of SHGs would lead to improved health-related behaviours of women in SHGs.

**Methods:**

A model for health layering of SHGs – *Parivartan* – was developed by the non-governmental organisation (NGO), Project Concern International, in 64 blocks of eight districts. Layering included health modules, community events and review mechanisms. The health layering model was adapted for use with government-led SHGs, called JEEViKA*+*HL, in 37 other blocks of Bihar. Scale-up of government-led SHGs without health layering (JEEViKA) occurred contemporaneously in 433 other blocks, providing a natural comparison group. Using Community-based Household Surveys (CHS, rounds 6-9) by CARE India, 62 reproductive, maternal, newborn and child health and nutrition (RMNCHN) and sanitation indicators were examined for SHGs with health layering (*Pavivartan* SHGs and JEEViKA+HL SHGs) compared to those without. We calculated mean, standard deviation and odds ratios of indicators using surveymeans and survey logistic regression.

**Results:**

In 2014, 64% of indicators were significantly higher in *Parivartan* members compared to non-members residing in the same blocks. During scale up, from 2015-17, half (50%) of indicators had significantly higher odds in health layered SHG members (*Parivartan* or JEEViKA+HL) in 101 blocks compared to SHG members without health layering (JEEViKA) in 433 blocks.

**Conclusions:**

Health layering of SHGs was demonstrated by an NGO-led model (*Parivartan*), adapted and scaled up by a government model (JEEViKA+HL), and associated with significant improvements in health compared to non-health-layered SHGs (JEEViKA). These results strengthen the evidence base for further layering of health onto the SHG platform for scale-level health change.

**Study registration:**

ClinicalTrials.gov number NCT02726230

Few interventions reach across the Sustainable Development Goals (SDGs) goals with the potential to address several SDGs simultaneously [1]. Self-help groups (SHGs) are a notable exception. In these groups, also known as women’s empowerment collectives and by a myriad of other names, women come together in groups of 10-20 for mutual aid and benefit. Groups are often formed for purposes of gaining access to credit or promoting livelihoods [2]. The women who participate in SHGs are typically low-income, mostly rural, and historically lack agency surrounding their own financial or health concerns [3]. By coming together for a common purpose, women in these groups, as Amartya Sen put it, counter the ‘feminisation of poverty’ [4]. SHGs often selectively engage the most marginalised communities, and thus have the potential to also improve equity [5,6]. Because of the varied form and structure of SHGs, their importance may extend well beyond purely financial and livelihood outcomes, leading to changes in health-related behaviours, shifting social norms and improving a wide range of health-related outcomes.

In India, SHGs started over 30 years ago with a direct tie to microfinance. SHGs have since expanded throughout India, including an estimated 200 million members in nine million SHGs; most maintain a goal of economic empowerment [7]. In 2006, the Bihar Rural Livelihood Program (BRLP) model – locally known as ‘JEEViKA,’ which means ‘livelihood’ in Hindi – was launched. JEEViKA is a government-run program largely funded by the World Bank that supports the formation and nurturing of SHGs based on microfinance and livelihoods promotion [8]. We call this SHG model “JEEViKA*,*” which did not have an explicit health component at the beginning of our evaluation in 2012.

In 2011, the Bill and Melinda Gates Foundation (BMGF) funded the *Parivartan* (“Transformation” in Hindi) project, which was implemented starting in 2012 by the non-governmental organisation (NGO) Project Concern International (PCI). *Parivartan* engaged women as change agents at family and community levels with the strategic objective of influencing health, nutrition and sanitation knowledge, practices and behaviours among women of reproductive age from the most marginalised communities in the eight focus districts (comprised of 64 blocks) of BMGF’s *Ananya* pilot program in Bihar (**Figure 1**). The SHG intervention complemented supply side support to the Government of Bihar (GoB) provided by CARE India and the social and behaviour change communications (SBCC) of BBC Media Action. Parivartan was a pilot project to understand processes and benefits of layering health interventions – namely reproductive, maternal, newborn and child health and nutrition (RMNCHN) and sanitation interventions – onto the SHG platform which historically had been formed based on microfinance and livelihood interventions. Parivartan was phased out in 2015; however, the SHGs created through Parivartan were transitioned to the government-run JEEViKA program and health layering was expanded. Evaluation by Population Council showed encouraging results across most RMNCHN indicators for Parivartan [9].

**Figure 1:**
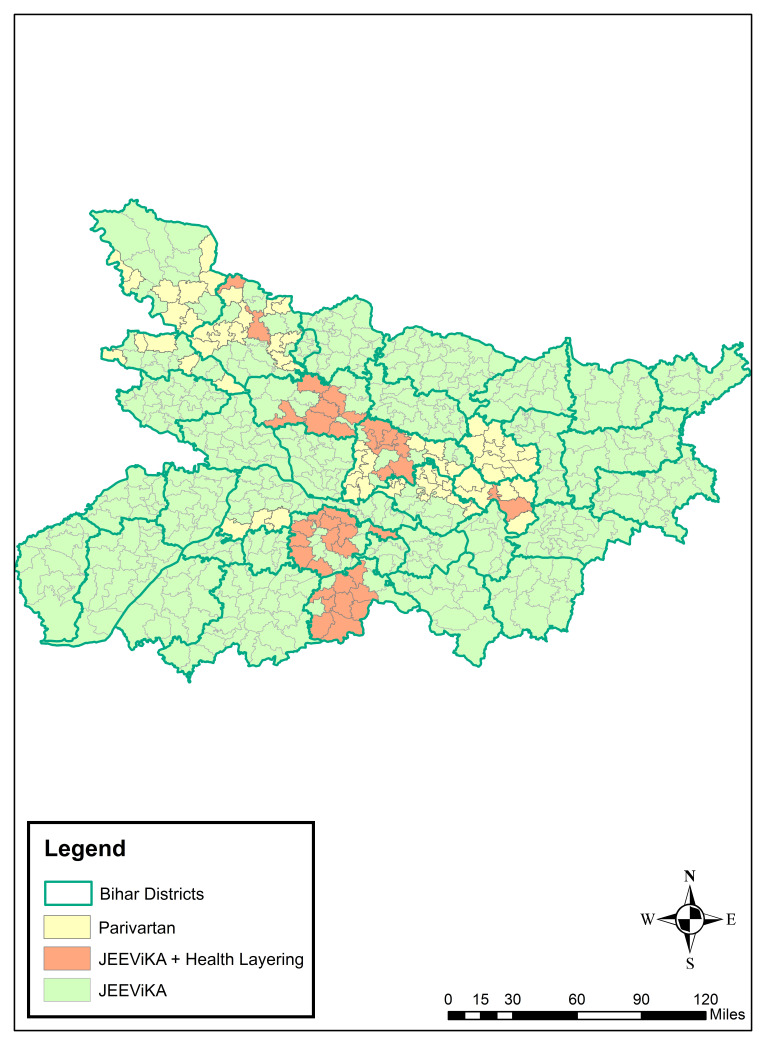
Map of Bihar showing self-help group types, 2014-2017.

To test the feasibility of implementing interventions similar to the NGO-led Parivartan model, but adapted for implemention among government-led JEEViKA SHGs, health layering was extended by PCI to 9089 JEEViKA SHGs in 37 additional blocks. Successful health layering of interventions in JEEViKA SHGs, creating JEEViKA+HL SHGs enhanced the interest of the GoB in scaling-up and leveraging community platforms to move the state’s health agenda forward. At the request of JEEViKA and the World Bank, PCI conceptualised the JEEViKA Technical Support Program (JTSP) in 2015 with the objective of providing support to the government to further scale up health layering in JEEViKA SHGs (**Figure 2**).

**Figure 2:**
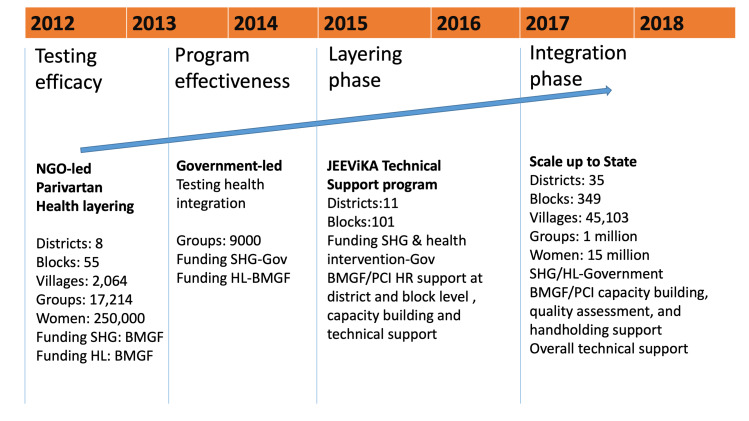
Timeline of health layering of self-help groups (SHGs) in Bihar, 2012-2018.

Despite increased global interest in health-layered SHG models, few evaluations have examined the health impacts of these interventions in relation to comparable, non-health-layered SHGs. Published reports poorly define health-layering and/or show mixed effects of health layering on a variety of health outcomes, such as maternal and child health, nutrition, gender violence, and mental health; in general, reports are lacking in aspects of quality in study design or a comparison group of SHG non-members [10-19].

The current study aims to address this evidence gap, building on preliminary promising results of the *Parivartan* intervention and capitalising on a natural comparison group, ie, non-health-layered JEEViKA SHGs [9,17,20]. Our analyses examine the impact of the NGO-led *Parivartan* model of health layering of SHGs, the impact of health-layering adapted for government-led SHGs during scale-up, and the isolated contribution of health layering by comparison with SHGs formed for purposes of microfinance and livelihoods promotion. We hypothesised that health-layered SHGs would have better performance on a broad range of RMNCHN indicators compared to non-health-layered SHGs.

## METHODS

### SHG Interventions

#### Health-layering of SHGs in the context of the *Ananya* program at scale

In order to examine the added effects of health layering on SHGs formed for promotion of livelihoods and access to credit, the strongest design would be a cluster randomised controlled trial comparing SHGs with or without health layering. A trial design was not feasible. Instead, we used a ‘natural’ separation of the groups with and without health layering in specific geographic blocks of Bihar, India and used these separations for an observational comparison.

SHG health-layered interventions were undertaken as part of a comprehensive suite of interventions funded by the BMGF in partnership with the GoB, as described previously [21,22]. The primary goal of the *Ananya* program was to strengthen the capacity of the GoB to improve RMNCHN outcomes state-wide. Several NGOs, including CARE India [23-28], BBC Media Action [29] and PCI [30] launched or supported the government in implementing a range of RMNCHN interventions during the *Ananya* pilot period of 2012 through 2013 in eight focus districts: Patna, Saharsa, East Champaran, West Champaran, Samastipur, Bengusarai, Gopalganj and Khagaria. During this pilot phase, PCI developed the *Parivartan* NGO-led health-layered SHG intervention in these eight districts (64 blocks). The *Parivartan* project officially started in November 2011 and implementation began in early 2012. In 2015, *Parivartan* groups were phased out and SHGs were scaled-up by the GoB statewide across all 38 districts. During scale-up, BMGF funded PCI to apply learning from *Parivartan* and provide technical support through the JTSP to health layering of government-led SHGs in the 64 blocks of the eight focus districts. The JEEViKA Technical Support Program (JTSP) also helped to promote health layering of former and newly formed JEEViKA groups in 37 additional blocks in three additional districts (**Figure 1**). Overall, by 2015 there were health-layered SHGs in 101 blocks of Bihar, while government-led JEEViKA SHGs without health layering were scaled up across the rest of the 433 blocks in the state.

#### Parivartan NGO-led health-layered SHGs: demonstration of the model

*Parivartan* formed new SHGs in marginalised communities comprised exclusively of Scheduled Tribes or Scheduled Castes (Hindu) or Pashmunda Muslims; some SHGs integrated Scheduled Tribes and Scheduled Castes together. *Parivartan* facilitated mutual learning and collective action, including Participatory Learning and Action (PLA), social mobilisation and empowerment, SBCC interventions and health education modules layered onto SHGs newly formed for microfinance and livelihoods promotion. Eleven health modules were layered upon NGO-led SHGs covering antenatal care, birth preparedness, postpartum and postnatal care, exclusive breastfeeding, complementary feeding, immunisations and sanitation behaviours (**Table 1**). Thus, “health layering” included promotion of health, nutrition and santitation behaviours across the RMNCHN continuum of care. Women were taught key health messages; empowered to develop confidence, self-esteem, and self-agency as well as social cohesion and collective action; and enabled to advocate for improvements in the quality of health services and to influence key health, nutrition and sanitation behaviours in their households and communities [9,20,31,32]. *Sahelis* (“female friends” in Hindi) were village-based volunteers who were trained to facilitate the adoption of improved behaviours through weekly educational and game-based sessions, and complementary activities such as accompanying women to antenatal and postnatal care visits. *Sahelis* were often younger women from similar communities with an expressed interest in health and had vocational training but not formal training in health and were supervised by officials as part of a tiered SHG structure called the Village Organisation. The intervention emphasised a linkage to existing village-level organisations and activities such as the Village Health, Sanitation and Nutrition Days (VHSNDs) and the work of CARE India and BBC Media Action to strengthen the subcentre health platform and the work of frontline workers (FLWs) [ie, Accredited Social Health Activists (ASHAs), Anganwadi Workers (AWWs), and Auxilliary Nurse Midwives (ANMs)] [23-26]. The *Parivartan* model for health layering upon NGO-led SHGs was developed in approximately 18 000 newly formed SHGs from 2012-2014 in 64 blocks during the eight-district pilot phase of *Ananya*, as described previously [21,22].

**Table 1:** Interventions in *Parivartan* health layering*

Session	Intervention content focus	Anticipated learning of self-help groups (SHGs)	Mode of delivery
1	Introductory module	Interrelation between health and livelihood	Banner with key messages and story of two women who had to invest a loan amount on a health emergency
			Consent letter by the SHG to continue the discussion on health, nutrition and sanitation
2	Antenatal care (ANC) and birth preparedness	Early registration for ANC	Message card and story of a Musahar pregnant lady
		Receipt of iron-folic acid tablets	
		Delivery in an institution	
3	Postnatal care. Focus of this module on early breastfeeding and neonatal behaviors like delayed bath, skin-to-skin care and dry cord care	Early initiation of breast feeding	Story of a lady named Sarita who has just delivered
		Not applying anything to the cord	
		Delaying bath for at least 72 hours	
		Practice skin-to-skin care	
4	Exclusive breastfeeding and supplementary nutrition	Exclusive breastfeeding for at least 6 months	Message card and picture puzzle card
		Children above 6 months given cereal based semi-solid food	
5	Routine immunization	Children receive appropriate doses of intervention according to schedule	Banner with key messages and song, a Sohar, a song which is traditionally Bihari and sung at the birth of a child
		Children complete DPT-3	
6	Family planning	Women use postpartum contraception	Story card with pictures
		Women continue to use contraception to prevent unintended pregnancies	
7	Personal hygiene and safe storage of water for the household	Decrease water borne disease	Picture cards and song
		Safe storage of water at household level	
		Handwashing at critical times	
8	Use of toilet and garbage management	Use of toilet	Faeces mapping(places used for open defacation were marked in yellow and then visualized so that community members could see all places at risk for contamination.
		Safe disposal of child’s stool	Picture card and song

DPT – diphtheria-pertussis-tetanus

*Adapted with permission from Saggurti et al., 2018 [9].

#### Layering of health into JEEViKA (JEEViKA+HL) SHGs for scaling

During the scale-up phase of *Ananya* beginning in 2014, the *Parivartan* intervention was phased-over to JEEViKA, with technical support from the JTSP and funding from the World Bank and the GoB. JEEViKA assumed the management of health-layered SHGs formed under *Parivartan*, and operationalised a tiered system of GoB-led SHGs starting with community mobilisation for women, then arranging them into SHGs, which were in turn aggregated into Village-level Organisations. These were further aggregated to clusters of federations at sub-block level [30]. Additional groups were scaled-up in the original 64 *Parivartan* blocks through the formation of new health-layered JEEViKA+HL SHGs. In an additional 37 blocks, capacity building of groups originally formed under JEEViKA to promote microfinance and livelihoods was also undertaken to layer in health, nutrition and sanitation programming under the JTSP. Thus, by 2015 a total of 101 out of 534 blocks in Bihar had approximately 150 000 health-layered SHGs [33]. The role of the *Sahelis* was replaced by JEEVIKA’s own grassroots cadre of community mobilisers who were trained on RMNCHN and sanitation messages by PCI.

#### Non-health-layered JEEViKA SHGs at scale

Non-health-layered JEEViKA SHGs in 433 other blocks throughout Bihar – separate from the 101 blocks where health layering of SHGs occurred – used the same tiered JEEViKA organisational structure and had an explicit credit and livelihood or agricultural focus, but did not include specific health, nutrition or sanitation interventions in 2016. The types of groups and their spread and scale-up can be viewed on the World Bank portal: https://arcg.is/051m4X.

### Evaluation

#### Community-based Household Surveys

To monitor progress of implementation of the *Ananya* program, CARE India undertook a series of Community-based Household Surveys (CHS) using a Lot Quality Assurance Sampling (LQAS)-like methodology, as described previously [23,34]. In the context of SHGs, the CHS surveys can be viewed as an independent data source as data were collected by CARE India’s Concurrent Measuring and Learning unit which was functionally independent of implementation. Questions to women in these surveys regarding their participation in SHGs started in survey round 6 in 2014 and continued through round 9 in 2017. In rounds 6-9 of the CHS, the methodology of survey administration and calculation of survey weights followed LQAS+ methodology in all 38 districts as described previously [23,34]. Within districts, the sampling frame was constructed within blocks from area Anganwadi Centers (AWCs) which are village-level institutions that provide basic education and nutrition services. From each selected AWC catchment area, eligible households were defined as containing a mother with a live birth and a young child in one of five age groups (0-2, 3-5, 6-8, 9-11 and 12-23 months). For each block, the sample size was proportionate to the known population of the block, subject to a minimum of 19 households. Women whose children had died in early childhood were not included. Data collection teams from CARE India went to each randomly selected household and administred surveys specific for mothers who had a child in one of five age groups to enable assessment of age-specific indicators across multiple domains of the continuum of care for health, nutrition and sanitation. This provided a sample size of 15 687, across all districts of the state, for each of the five age groups, for each CHS survey.

CHS survey questions about particiption in SHGs were utilised to identify women who self-identified as being a member of an SHG. To define the type of SHG to which women belonged, information available on the timing of implementation of SHGs in specific blocks in Bihar was used. The *Parivartan* program was implemented in 64 blocks in the eight original focus districts of *Ananya;* at that time the governmental JEEViKA program was not yet active in these blocks. Thus, if a woman surveyed at the time of CHS round 6 (mid-2014) said she was a member of a SHG and lived in one of these 64 blocks, we assigned her to the SHG type *Parivartan*. Age-comparable women residing in the same blocks but who did not report membership in an SHG served as the comparison group for assessment of impacts of *Parivartan* health-layered SHGs. During round 6 in 2014, no health layering of SHGs was occurring in the other 469 blocks in Bihar.

At the time of CHS rounds 8 and 9 (2016-2017), groups in the former *Parivartan* health layered blocks (n = 64) had been transitioned to the government to manage or were newly formed government-managed groups into which health layering had occurred; we identified both of these types of groups as JEEViKA+HL. Also in rounds 8 and 9, 37 additional blocks engaged in health layering upon existing government groups; women in those blocks who self-identified as SHG members at that time were defined as belonging to JEEViKA+HL groups. Although they had different origins, the health-layered groups in all 101 blocks shared a common JEEViKA management structure in rounds 8 and 9. The rest of the blocks of the state (n = 433) were considered non-health-layered JEEViKA groups, as regular SHGs without specific health layering were predominant throughout the rest of the state during this time period.

Using this information, we were able to separate women into three types of SHGs: 1) Health-layered NGO-led *Parivartan* SHGs funded by the BMGF and led by PCI in 64 blocks from 2011-2014 (assessed in CHS round 6); 2) Health-layered JEEViKA+HL groups coming from two sources: a) the original 64 *Parivartan* blocks where health-layered *Parivartan* SHGs transitioned in 2014-2015 to JEEViKA+HL SHGs which were managed under the JTSP, and b) 37 additional blocks where health was layered into JEEViKA SHGs, for a total of 101 blocks with JTSP-managed JEEViKA+HL SHGs in 2015-2017 (assessed in CHS rounds 8-9 in 2016-2017); and 3) Non-health-layered JEEViKA SHGs in the state’s remaining 433 blocks in 2015-2017 (assessed in CHS rounds 8-9 in 2016-2017).

#### Indicator selection and categorisation

We focused on 62 indicators which broadly reflected the original health, nutrition and sanitation modules developed for *Parivartan* SHGs and adapted for scale-up through JEEViKA*+*HL SHGs (**Table 2**). We restricted our analytical cohort to women with children aged 0-2 months for antenatal, delivery, postnatal newborn and postpartum family planning indicators; 9-11 months for immunisation and complementary feeding indicators, and 12-23 months for family planning and sanitation. These indicators were selected because they were most relevant to the health modules layered upon SHGs and represented a broad array of indicators across the RMNCHN continuum of care and delivery platforms.

**Table 2:** Reproductive, maternal, newborn and child health, nutrition and sanitation indicators by continuum of care domain and delivery platform from Community-based Household Surveys

	Indicator	Continuum of care domain	Delivery platform	Variable name	Age group
1	4+ ANC visits	Antenatal care	Facility/outreach service delivery	r_fouranc	0-2
2	Had at least one ANC exam if reporting any ANC visit	Antenatal care	Facility/outreach service delivery	Anyancexam	0-2
3	Admitted to hospital for complication	Antenatal care	Facility/outreach service delivery	admitcompl	0-2
4	Received at least 90 IFA tablets during pregnancy	Antenatal care	Facility/outreach service delivery	gotifa90	0-2
5	FLW antenatal home visit to discuss mother's or baby’s health	Antenatal care	Frontline worker performance	flwvishlth	0-2
6	Any FLW visit during last trimester	Antenatal care	Frontline worker performance	any_flw_3rdtrim	0-2
7	FLW advised on hand-washing by delivery attendant	Antenatal care	Frontline worker performance	advice_hand	0-2
8	FLW advised on danger of excessive bleeding	Antenatal care	Frontline worker performance	advice_bleed	0-2
9	FLW advised on danger of convulsions	Antenatal care	Frontline worker performance	advice_conv	0-2
10	FLW advised on danger of prolonged or difficult labor	Antenatal care	Frontline worker performance	advice_labor	0-2
11	FLW advised on danger of swelling of face or hands	Antenatal care	Frontline worker performance	advice_edema	0-2
12	FLW advised on reasons to deliver in a hospital	Antenatal care	Frontline worker performance	advice_facdel	0-2
13	FLW advised on saving money in case of emergency	Antenatal care	Frontline worker performance	advice_money	0-2
14	FLW advised on pregnancy danger signs	Antenatal care	Frontline worker performance	advice_preg_signs	0-2
15	Consumed 90+ IFA tablets	Antenatal care	Mother's behaviour	tookifa90	0-2
16	Pregnancy registration in the first trimester	Antenatal care	Mother's behaviour	pregreg1sttrim	0-2
17	Sought care for complications	Antenatal care	Mother's behaviour	r_careseeking_compl	0-2
18	Saved money	Antenatal care	Mother's behaviour	savemoney	0-2
19	Chose a facility for delivery	Antenatal care	Mother's behaviour	pickfac	0-2
20	Chose a facility in case of emergency	Antenatal care	Mother's behaviour	pickemfac	0-2
21	Arranged transportation to facility	Antenatal care	Mother's behaviour	pickvehicle	0-2
22	Delivery in a facility (public or private)	Delivery	Facility/outreach service delivery	pod_facility	0-2
23	Delivery in a private facility (out of all deliveries)	Delivery	Facility/outreach service delivery	privatefac	0-2
24	Delivery in a public facility (out of all deliveries)	Delivery	Facility/outreach service delivery	publicfacoffac2	
25	Caesarian-section for delivery	Delivery	Facility/outreach service delivery	Csection	0-2
26	New blade was used to cut cord	Delivery	Facility/outreach service delivery	new_blade	0-2
27	Clean cloth was used for baby	Delivery	Facility/outreach service delivery	clean_cloth	0-2
28	Clean thread was used to tie cord	Delivery	Facility/outreach service delivery	clean_thread	0-2
29	Baby weighed at birth	Delivery	Facility/outreach service delivery	r_weighed	0-2
30	Baby immediately dried and wrapped	Delivery	Mother's behaviour	dried_wrapped	0-2
31	Any FLW visits in the first week after delivery	Postnatal care	Frontline worker performance	any_flw_visit_1stweek	0-2
32	3+ FLW visits in the first week after delivery	Postnatal care	Frontline worker performance	three_flw_visit_1stweek	0-2
33	FLW advised on neonatal danger signs	Postnatal care	Frontline worker performance	advice_neonatal_signs	0-2
34	FLW advised on delayed bathing	Postnatal care	Frontline worker performance	advice_delaybath	0-2
35	FLW advised on skin-to-skin care	Postnatal care	Frontline worker performance	advice_stsc_new	0-2
36	FLW advised on dry cord care	Postnatal care	Frontline worker performance	advice_dry_cord	0-2
37	Skin-to-skin care	Postnatal care	Mother's behaviour	stsc_imm_later	0-2
38	Dry cord care	Postnatal care	Mother's behaviour	drycordcare3	0-2
39	Delayed bath	Postnatal care	Mother's behaviour	delay_bath	0-2
40	Care seeking for neonatal complications	Postnatal care	Mother's behaviour	r_careseeking_newborn	0-2
41	FLW advised on early initiation of breastfeeding	Nutrition	Frontline worker performance	advice_bf_pre	0-2
42	FLW advised on exclusive breastfeeding	Nutrition	Frontline worker performance	advice_exc_bf	0-2
43	FLW advised on age to which to continuing breastfeeding	Nutrition	Frontline worker performance	advice_age_bf	0-2
44	Immediate breastfeeding	Nutrition	Mother's behaviour	r_bf1	0-2
45	Exclusive breastfeeding in the past 24 hours	Nutrition	Mother's behaviour	EBF_last24hrs	0-2
46	Initiation of complementary feeding	Nutrition	Mother's behaviour	initiate_CF	9-11
47	Age-appropriate initiation of complementary feeding (6-8 months of age)	Nutrition	Mother's behaviour	age_initiate_cf	9-11
48	Age-appropriate frequency of complementary feeding (3+ times for 9-11 month-old children)	Nutrition	Mother's behaviour	age_approp_freq_cf	9-11
49	Fed complementary cereal-based food in past 24 hours	Nutrition	Mother's behaviour	Cereal	9-11
50	FLW reminded on vaccine information	Immunisation	Frontline worker performance	advice_remind_vaccine	0-2
51	Have immunisation card	Immunisation	Facility/outreach service delivery	have_immcard	9-11
52	Polio (OPV3 or IPV) by card	Immunisation	Facility/outreach service delivery	polio3_card	9-11
53	DPT3 by card	Immunisation	Facility/outreach service delivery	dpt3_card	9-11
54	FLW asked interest in having more children	Family planning	Frontline worker performance	advice_askfp	0-2
55	FLW asked risk of becoming pregnant post-delivery	Family planning	Frontline worker performance	advice_pregrisk	0-2
56	FLW advised on sterilization post-delivery	Family planning	Frontline worker performance	advice_pptl	0-2
57	FLW advised on use of PPIUD post-delivery	Family planning	Frontline worker performance	advice_pptiud	0-2
58	Modern method of contraception used	Family planning	Mother's behaviour	use_modernfp	9-11
59	Washed hands before feeding child	Sanitation	Mother's behaviour	Washafterfeed	9-11
60	Washed hands after using toilet	Sanitation	Mother's behaviour	Washaftertoil	9-11
61	Used soap or detergent when washing hands before feed	Sanitation	Mother's behaviour	WAFuns	9-11
62	Used soap or detergent when washing hands after toilet	Sanitation	Mother's behaviour	WATuns	9-11

ANC – antenatal care, CHS – Community-based Household Survey, DPT – diphtheria-pertussis-tetanus, FLW – frontline worker, IFA – iron-folic acid, IPV – inactivated polio vaccine, OPV – oral polio vaccine, PPIUD – postpartum intrauterine device, SHG – self-help group

Prior to analysis, indicators were grouped into the following domains according to the continuum of care, as described previously [28]: antenatal care and birth preparedness, delivery (childbirth care), postnatal care, nutrition/complementary feeding, immunisation, family planning and sanitation. For each continuum-of-care domain, we further classified the indicators into one of three delivery platforms: FLW performance or behaviour, mother’s behaviour, and facility care or outreach service delivery, as described previously [32].

### Statistical analysis

We examined demographic characteristics for SHG-member and non-member women in *Parivartan* blocks (n = 64), JEEViKA*+*HL blocks (n = 37) and JEEViKA blocks (n = 433), separately. Then, we examined relevant comparisons by calculating odds ratios (ORs) and 95% confidence intervals (CIs) for all 62 indicators using survey logistic regression.

To understand SHG health layering effects according to the timeline of program interventions, we calculated one set of ORs for CHS round 6 (mid 2014) separately, as this timing was related to the *Parivartan* NGO-led health layering intervention in 64 blocks. To further understand the transition to scale up of government-led health-layered JEEViKA+HL SHGs, we calculated a second set of ORs for CHS rounds 8 and 9 (2016-2017) in 37 blocks. Lastly, to isolate the effects of health layering, we compared SHG members in *Parivartan* and JEEViKA*+*HL blocks (101 blocks with NGO-led and government-led health-layered SHGs, combined) compared to SHG members in non-health-layered JEEViKA blocks (433 blocks). In each of the abovementioned analyses, we calculated separate survey logistic regression models, deriving ORs and 95% CIs of the SHG term for each RMNCHN and sanitation indicator. Due to the large number of comparisons, we applied the False Discovery Rate (FDR) multiple comparison adjustment controlling procedure by Benjamini and Hochberg [35], applying an upward adjustment t*o the P values to control family-wise type I error, alpha, at 0.05.* We additionally adjusted all models for maternal age and focal child gender. For all models, a sensitivity adjustment for additional co-variates was performed, including household size, number of children, asset index, Hindu religion, Scheduled Tribe/Scheduled Caste, literacy level, whether the family lived with nuclear or extended family members, mother’s and father’s educational levels and whether the family lived in a Pucca (ie, solid, permanent, built of substantial material such as stone, brick, cement, concrete, or timber) house. As some of these adjustment variables were highly collinear with the primary predictor, SHG membership, and thus some models would not parameterise, we present data based on adjustment for maternal age and focal child gender only for our primary analyses. Analyses were reported in the form of forest plots according to RMNCHN and sanitation continuum-of-care domains.

### Ethical considerations

Permission for access and terms of CHS data use were agreed upon with CARE India through a data sharing agreement and approved by the Stanford University Institutional Review Board protocol #39719. This study is part of the BMGF Bihar program which was registered with ClinicalTrials.gov number NCT02726230.

### Role of the funding source

This study was supported by grants from the BMGF, including: OPP1163688 to Stanford University for analyses and manuscript preparation, OPP1033907 to PCI for SHG health layering, OPP1141832 to Population Council for SHG evaluation, and OPP1084426 to CARE India for CHS evaluations. BMGF India Country Office program officers reviewed the manuscript for accuracy and adequate description of the interventions, study design, and data collection. The senior author had full access to the data and independence from the funders in the reporting of results, the interpretation of the data and the decision to publish the manuscript.

## RESULTS

### Study population

Socioeconomic and demographic characteristics of the study population are displayed in **Table 3** and Table 4. Overall, compared to non-members, SHG members combined across the three SHG types (*Parivartan*, JEEViKA+HL, JEEViKA) across rounds 6-9 of CHS during 2014-2017 were slightly older, had more children, were more often from a Scheduled Caste and Hindu, less likely to have formal education/literacy, and had poorer living conditions (ie, less likely to dwell in a ‘pucca’ house) and a lower average number of household assets. Thus, SHG member women were, in general, at greater social disadvantage or more marginalised than non-SHG members. This was by design, as *Parivartan* and JEEViKA targeted the formation of SHGs among the most marginalised women.

**Table 3:** Demographic characteristics of self-help group (SHG) members across various block groups, Community-based Household Surveys rounds 6-9 combined

	SHG members in 37 JEEViKA+HL blocks			SHG members in 433 JEEViKA blocks			SHG members in 64 *Parivartan* blocks			Non-SHG members in 37 blocks			Non-SHG members in 433 JEEViKA blocks				Non-SHG member in 64 *Parivartan* blocks			
**Variable**	**N**	**Median**	**IQR**	**N**	**Median**	**IQR**	**N**	**Median**	**IQR**	**N**	**Median**	**IQR**		**N**	**Median**	**IQR**		**N**	**Median**	**IQR**	
Age of mother	840	24.4*	21.7-27.3	7718	24.4*	21.6-27.5	1377	24.5*	21.6-27.6	3298	22.4	19.9-24.9		43 788	23.0	20.4-25.6		5669	22.7	20.1-25.3	
Household size	840	5.9*	4.6-8.2	7718	6.0*	4.5-8.1	1377	5.9*	4.6-7.8	3298	6.6	4.7-9.1		43 788	6.6	4.7-9.0		5669	6.6	4.7-9.0	
Number of children in household	840	2.5*	1.5-3.6	7718	2.6*	1.6-3.7	1377	2.7*	1.6-3.9	3298	1.6	1.0-2.7		43 788	1.7	1.0-2.9		5669	1.7	1.0-2.9	
Number of adults in household	840	2.1*	1.3-5.3	7718	2.0*	1.3-5.0	1377	1.9*	1.3-4.5	3298	4.0	1.7-7.0		43 788	3.9	1.6-6.9		5669	4.0	1.7-6.9	
Mother years of education	840	0	0-5.6	7718	0	0-6.0	1377	0	0-4.3	3298	1.5	0-8.5		43 788	0	0-8.3		5669	0	0-7.6	
Father years of education	806	4.0*	0-8.1	7443	2.3*	0-7.9	1323	0*	0-7.3	3177	5.3	0-9.4		42 306	5.6	0-9.3		5478	4.7	0-9.0	

HL – health layering, N – sample size, IQR – interquartile range

*Significant difference between SHG members and and non-SHG members in the indicated block group, *P* < 0.01.

**Table 4:** Demographic characteristics of self-help group (SHG) members across various block groups, Community-based Household Surveys rounds 6-9 combined

		SHG members in 37 JEEViKA+HL blocks			SHG members in 433 JEEViKA blocks			SHG members in 64 *Parivartan* blocks			Non-SHG members in 37 blocks			Non-SHG members in 433 JEEViKA blocks		
**Variable**		**Frequency**	**Row %**	**Coeff. of variation**	**Frequency**	**Row %**	**Coeff. of variation**	**Frequency**	**Row %**	**Coeff. of variation**	**Frequency**	**Row %**	**Coeff. of variation**	**Frequency**	**Row %**	**Coeff. of variation**
Focal child	Female	408	48.80*	0.04	3692	47.6*	0.0	699	51.04*	0.03	1612	48.63	0.02	20 923	47.8	0.0
	Male	432	51.20*	0.04	4026	52.4*	0.0	678	48.96*	0.03	1686	51.37	0.02	22 865	52.2	0.0
Nuclear	No	451	50.80*	0.04	3870	49.6*	0.0	638	46.77*	0.03	2321	67.47	0.01	28 968	66.1	0.0
	Yes	389	49.20*	0.04	3848	50.4*	0.0	739	53.23*	0.03	977	32.53	0.03	14 820	33.9	0.0
Literacy	No	524	61.24*	0.03	4921	63.8*	0.0	943	67.68*	0.02	1688	49.30	0.02	23 425	53.0	0.0
	Yes	316	38.76*	0.05	2797	36.2*	0.0	434	32.32*	0.04	1610	50.70	0.02	20 363	47.0	0.0
Literacy husband	No	354	42.59*	0.04	3569	46.6*	0.0	680	48.55*	0.03	1219	36.19	0.03	16 707	37.8	0.0
	Yes	485	57.41*	0.03	4138	53.4*	0.0	693	51.45*	0.03	2071	63.81	0.01	27 023	62.2	0.0
Caste	General/Other	35	4.22*	0.18	462	5.7*	0.0	53	3.41*	0.16	339	9.81	0.06	5509	12.9	0.0
	OBC	468	56.42*	0.03	4340	55.8*	0.0	813	58.16*	0.03	1955	60.93	0.02	27 101	61.6	0.0
	Scheduled caste	321	37.52*	0.05	2756	36.4*	0.0	491	36.99*	0.04	945	27.65	0.03	10 301	23.5	0.0
	Scheduled tribe	16	1.84*	0.26	160	2.2*	0.1	20	1.44*	0.23	59	1.61	0.14	877	2.1	0.0
Religion	Buddhist	0						0			0			1	0.0	1.0
	Christian	1	0.11*	1.00	4	0.1*	0.5	0	.	.	4	0.12	0.53	47	0.1	0.2
	Hindu	795	94.19*	0.01	6806	87.6*	0.0	1247	90.55*	0.01	2992	89.81	0.01	36 837	83.0	0.0
	Jain	0			1	0.0	1.0	0	.	.	1	0.04	1.00	3	0.0	0.6
	Muslim	44	5.70*	0.16	906	12.3*	0.0	130	9.45*	0.09	301	10.03	0.06	6886	16.9	0.0
	Other	0			1	0.0	1.0	0			0			8	0.0	0.4
	Sikh	0			0			0			0			1	0.0	1.0
Hindu/SCST	Hindu, non-scst	461	55.20*	0.03	3919	49.4*	0.0	744	52.52*	0.03	2000	60.90	0.02	25 879	58.0	0.0
	Hindu, scst	334	38.99*	0.05	2887	38.2*	0.0	503	38.03*	0.04	992	28.90	0.03	10 958	25.0	0.0
	Non-hindu	45	5.81*	0.16	912	12.4*	0.0	130	9.45*	0.09	306	10.19	0.06	6946	17.0	0.0
Ghar	Kachcha	202	24.61*	0.06	2697	36.1*	0.0	467	34.10*	0.04	761	22.05	0.03	13 684	31.1	0.0
	Pucca	113	13.57*	0.09	735	8.9*	0.0	124	8.92*	0.09	683	20.01	0.04	7936	17.6	0.0
	Semi-Pucca	525	61.82*	0.03	4286	55.0*	0.0	786	56.98*	0.03	1854	57.94	0.02	22 168	51.4	0.0

HL – health layering, SCST - scheduled caste/scheduled tribe, SHG – self help group

*Indicates significant difference between SHG and non SHG comparison in the block group (433/37/64).

### Effects of *Parivartan* health-layered SHGs

**Figure 3:**
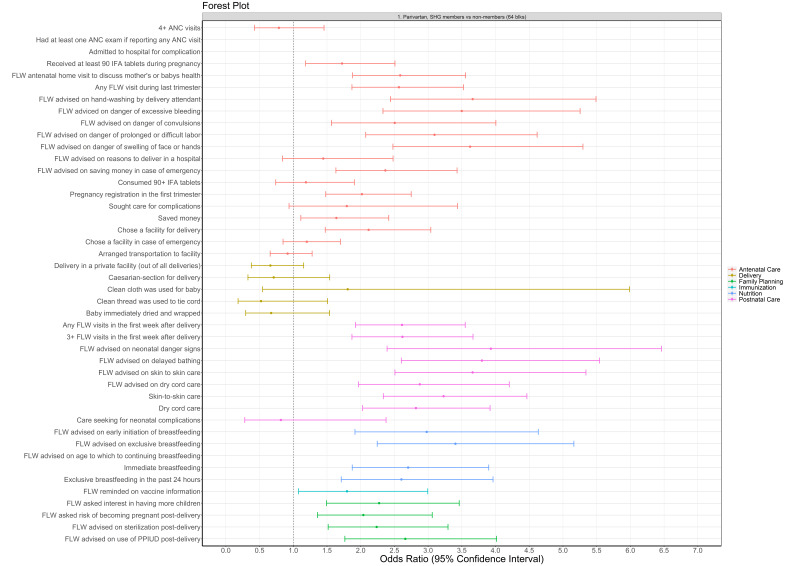
Effect of *Parivartan* health layering on reproductive, maternal, newborn and child health and nutrition indicators by continuum of care, CHS round 6 (2014), compared *to* non-members, 64 blocks (odds ratio, OR ± 95% confidence interval, CI?. All models presented were adjusted for age of the mother and the sex of the focal child. These models also accounted for the study’s complex design by applying study weights. ANC – antenatal care, CHS – Community-based Household Survey, DPT – diphtheria-pertussis-tetanus, FLW – frontline worker, HL – health layering, IFA – iron-folic acid, IPV – inactivated polio vaccine, OPV – oral polio vaccine, PPIUD – postpartum intrauterine device, SHG – self-help group (sanitation indicators were not included herein as the models did not parameterise).

Nearly two thirds (64%) of RMNCHN indicators which had been targeted through modules for health layering were significantly higher for SHG members compared to non-members in the 64 *Parivartan* blocks in 2014 (CHS round 6) (**Figure 3**; Table S1a in the **Online Supplementary Document**). Several key indicators were higher for *Parivartan* SHG members compared to non-members, including 2-fold higher pregnancy registration in the first trimester (OR = 2.02, 95% CI = 1.48-2.75), 3-fold higher skin-to-skin care (OR = 3.23, 95% CI = 2.3-4.5), and nearly 3-fold higher dry cord care (OR = 2.82, 95% CI = 2.03-3.9) and immediate breastfeeding (OR = 2.7, 95% CI = 1.9-3.9). Sanitation indicators were unstable in round 6 and were not reported. Nearly all (96%) FLW performance indicators had higher levels for SHG members than non-members in *Parivartan* blocks, nearly half (44%) of mother’s behaviour indicators had higher levels, and only one out of seven (14.2%) of the facility/outreach service delivery variables – receiving at least 90 IFA tablets during pregnancy (OR = 1.7, 95% CI = 1.2-2.5) – had higher odds for SHG members in *Parivartan* blocks.

### Effects of JEEViKA+HL health-layered SHGs

In the 37 JEEViKA*+*HL blocks, over one quarter (27%) of indicators had positive ORs for SHG members compared to non-members in rounds 8-9 (**Figure 4**, center; Table S1b in the **Online Supplementary Document**). One quarter (25%) of antenatal, 22% of postnatal, 40% of nutrition, and 75% of family planning indicators were significantly higher for SHG members compared to non-members. Delivery, immunisation and sanitation indicators were similar for SHG members and non-members. Less than 5% of indicators were worse for SHG members. In terms of delivery platform, 32% of FLW performance indicators, 33% of mother’s behaviour indicators, and 0% of the facility/outreach service delivery indicators had higher odds for SHG members than non-members in the 37 JEEViKA*+*HL blocks.

### Effects of non-health-layered JEEViKA *SHGs*

In the 433 non-health-layered JEEViKA blocks, 50% of RMNCHN indicators were significantly higher for SHG members compared to non-members in rounds 8-9 (**Figure 4**, right; Table S1c in the **Online Supplementary Document**). Across the continuum of care, 60% of antenatal, 44% of postnatal, 75% of nutrition, and all family planning indicators were higher for SHG members. Immunisation, delivery and sanitation indicators were similar for SHG members and non members. Overall, 12.5% of indicators were worse for SHG members in JEEViKA blocks.

#### JEEViKA+HL compared to JEEViKA SHGs

**Figure 5:**
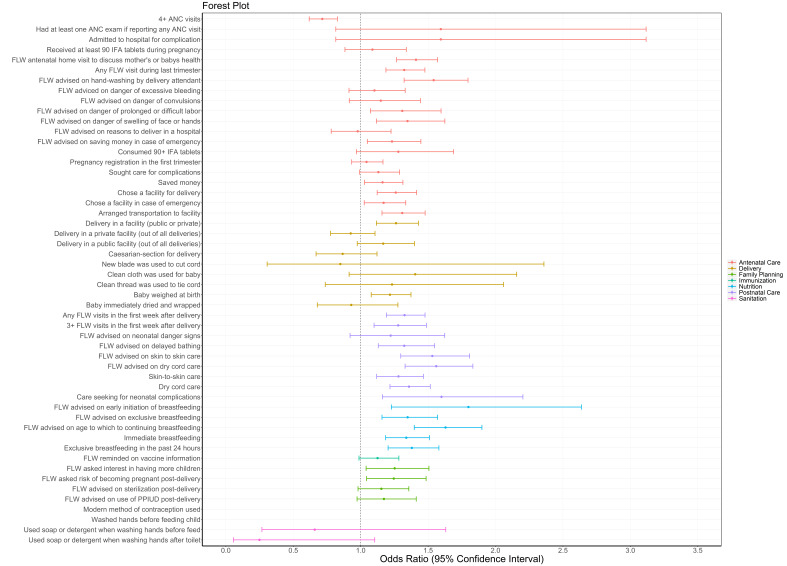
Effect of SHG interventions on reproductive, maternal, newborn and child health, nutrition and sanitation indicators by continuum of care, comparing members in health-layered SHGs (JEEViKA+HL and *Parivartan*) to members in non-health-layared JEEViKA SHGs, CHS rounds 8-9, 2016-2017. All models presented were adjusted for age of the mother and the sex of the focal child. These models also accounted for the study’s complex design by applying study weights. ANC - antenatal care, CHS – Community-based Household Survey, DPT - diphtheria-pertussis-tetanus, FLW – frontline worker, HL – health layering, IFA – iron-folic acid, IPV – inactivated polio vaccine, OPV – oral polio vaccine, PPIUD – postpartum intrauterine device, SHG – self-help group.

When comparing the performance of SHG members in the 101 blocks with health layering (64 *Parivartan* and 37 JEEViKA*+*HL blocks) to the performance of SHG members in 433 non-health-layered JEEViKA blocks in CHS rounds 8-9 from 2016-2017, half of indicators (50%) had significantly higher odds in the health layered JEEViKA+HL groups compared to non-health-layered JEEViKA groups (**Figure 5**, Table S2 in the **Online Supplementary Document**). Health-layered SHG members showed significantly higher odds for half (50%) of antenatal care, one fourth (22%) of delivery, a majority (88%) of postnatal, 55% of nutrition, 33% of family planning and no sanitation indicators. According to delivery platform, 70% of FLW performance indicators, 41% of maternal behaviour indicators, and 17% of facility/outreach service delivery indicators had significantly higher odds for SHG members with health layering compared to SHG members without health layering.

## DISCUSSION

**Figure 6:**
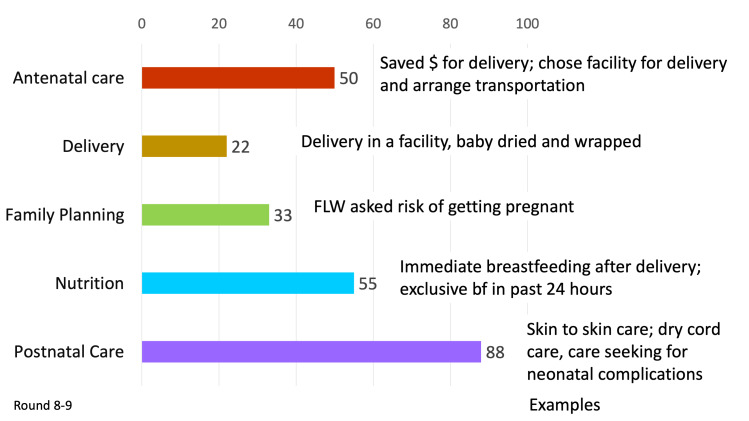
*Summary* of SHG interventions on reproductive, maternal, newborn and child health and nutrition domains, comparing members in health-layered SHGs (JEEViKA+HL and *Parivartan*) to members in non-health-layared JEEViKA SHGs, CHS rounds 8-9, 2016-2017. (% of indicators where HL outperformed SHG alone). Sanitation indicators are not included here as sanitation indicators had similar effects in JEEViKA+HL and JEEViKA groups.

SHGs are a powerful vehicle for health promotion for women and young children. Our study utilised repeated, cross-sectional data to demonstrate improvements across a range of RMNCHN behaviours among members of health-layered SHGs. Our original hypothesis, that health-layered SHGs would outperform non-health-layered SHGs held true, particularly for antenatal and postnatal care and nutrition, and less so for family planning, delivery care or sanitation (**Figure 6**). This expands the evidence for impact of microcredit and livelihood-focused SHGs, which have been studied in randomised controlled trials, [36] quasi-experimental [37,38] and observational studies [39,40], and have shown variable impacts on health. With focused health promotion within these SHGs, significant improvements in RMNCHN behaviours can be achieved.

This study demonstrated broad health impacts of NGO-led health layering of SHGs, as well as the feasibility of adapting and transitioning health layering from an NGO-led to a government-led model. The model for health layering was developed by the NGO, PCI, implemented at substantial scale in 64 blocks of Bihar, and showed significantly higher levels of approximately two-thirds of RMNCHN indicators in SHG members compared to non-members. Given the success with health layering, the JTSP was formed with PCI leadership to provide technical support to the government to layer health promotion onto government-led JEEViKA SHGs across 37 blocks with more modest impact, ie, approximately one-quarter of RMNCHN indicators were significantly higher among women members of JEEViKA+HL SHGs compared to non-members. Effects were most evident across the domains of antenatal care, birth preparedness, postnatal care and family planning, but were weaker for delivery care, complementary feeding/nutrition, sanitation and immunisation indicators. These results demonstrated the feasibility of transferring an NGO-developed model for health promotion to government-led SHGs at scale, although the findings also reveal the potential to further improve the health impact of government-led health-layered SHGs.

Finally, we showed that health layering of government-led SHGs (JEEViKA+HL) produced additional health benefits, particularly for antenatal care, postnatal care, nutrition and family planning beyond that seen for JEEViKA SHGs formed primarily for access to credit and livelihood promotion. Overall, 50% of indicators measured performed better in SHG members in 101 blocks with health-layered JEEViKA+HL SHGs compared to JEEViKA SHGs without health layering, despite the fact that non-health-layered SHGs had substantial impacts in improving health, as demonstrated previously [30]. Literature examining health-layered compared to non-health-layered SHGs is weak; despite a plethora of reviews, the quality of evidence appears to be low [16,17], although some members of our team are now conducting a systematic review to comprehensively assess the evidence for SHG health layering. A new trial on the effects of participatory learning and action meetings layered upon SHGs is currently under way to examine nutritional outcomes, including complementary feeding, and dietary diversity and composition [41].

A novel finding in our study is that health layering was accomplished effectively at scale through government-led groups. Moreover, we documented effect sizes of government-led health-layered SHGs at scale of about the same magnitude as seen in prior reports of smaller-scale NGO-led pilot studies [9,20], suggesting that governmental scale-up of health-layered SHGs under technomanagerial support of the JTSP was effective in promoting health improvements. While some *Ananya*-based interventions saw significant changes, the majority of non-SHG *Ananya* interventions showed more leveling of RMNCHN indicators during scale-up under the Bihar Technical Support Program, a similar support structure for non-SHG RMNCHN interventions [22,28]. In sharp contrast, SHG health layering had positive health benefit through the scale-up phase. The reasons for these positive effects may be that this government-led program operates through largely automous structures and functions – including human resources and performance management, largely funded by World Bank loans – and with strong political support and JEEViKA program leadership. This has ensured steady access to resources and committed and capable program staff at all levels.

Our findings also suggest that fertile ground exists in Bihar for further health-layering of SHGs statewide, which may have an added impact on health equity. Our previous work suggests large variation in equity, and health promotion upon SHGs may be a way to address these large differences [42]. SHG members in non-health-layered JEEViKA blocks performed better in half of RMNCHN indicators than age-comparable non-SHG-members residing in the same blocks, demonstrating substantial benefits of group membership and the potential for further health layering. For all three types of SHGs (*Parivartan*, JEEViKA+HL, JEEViKA), improvements in RMNCHN indicators were seen compared to women who were not in groups [30], in spite of the observation that women in groups were more highly marginalised than non-group members. Given that the women targeted by SHGs are more marginalised – on average having more children, less education, and greater levels of poverty – the positive effects of health layering are even more remarkable.

The findings regarding the impacts of SHGs on specific domains of health largely corroborated earlier findings by Saggurti et al. [9,20], showing similar increases in skin-to-skin care, timely initiation of breastfeeding, exclusive breastfeeding, and increased use of modern contraception/family planning. Less impacts were seen on facility-based deliveries, similar to our prior work for women in Bihar in general [27]. However, our study extends this prior research as we captured a full range of RMNCHN and sanitation indicators over the first one thousand days of a child’s life. It may be that underpinning SHG effectiveness are innate human evolutionary bonding mechanisms, where women during/after pregnancy value the advice from older women, which they are likely to uptake. Drawing upon this, it is also conceivable that benefits of SHG participation extended even beyond the activities measured in this evaluation. Mutual synergies must be explored across sectors in future research. For example, layering health upon SHGs formed for women’s economic empowerment may lead to increases in other person-centered care across health, nutrition, sanitation and hygiene, and in health education, for example HIV awareness. Beyond this, there may be impacts related to individual and collective agency and action related to outcomes beyond health, such as gender-based violence and other gender-related or social issues. Recent reviews corroborate these findings that SHG membership positively affects several health indicators and behaviours, including neonatal [5] and maternal mortality [15,43], through “planned, do, study act cycles” [15,41,44,45] and for HIV indicators [10] and mental health [12,15]. Moreover, prior research has indicated that SHGs, specifically those linked to microfinance, increase non-health outcomes like financial outcomes, links to community health access, and health care financing/insurance [13,14,18,19]. Other reviews on SHGs suggest that impacts can be seen in agricultural SHGs [10], and that effects seen in older women can also be seen in adolescents [11].

Compared to other studies of SHG membership, this evaluation had several strengths. It 1) included a comparison group of non-SHG members to establish the feasibility of scaling up health-layered SHGs; 2) determined and compared SHG effects on a variety of indicators, across the RMNCHN continuum of care from pregnancy to early childhood and through several delivery platforms; and 3) examined specific contributions of health layering compared to SHG groups without health layering, showing an additional positive effect of health layering on RMNCHN and sanitation outcomes.

In addition to successful scaling of the SHG platform with added health layering upon this platform, there may be cyclic gains when SHG women gain empowerment, which potentially also acts as a lever for improving health, for example through increasing demand for services or accessing and connecting with the subcentre platform. This cyclic action derives from the conceptual theories of change that underlie the SHG program model. SHGs encompass a participant empowerment model, wherein SHG members engage in group actions which increase self-efficacy and women’s trust in the health system, and encourage women to have specific dialogues about their circumstances, thereby increasing the autonomy of women’s health as well as their collective efficacy and utilisation of health services. Close access to primary health care has been linked to improved health outcomes such as lower childhood mortality, substantiating the importance of providing health services locally in a manner that is both acceptable and impacts women (Irani L, unpublished results).

Our evaluation had some limitations. First, this study did not utilise an experimental design and thus, confounders which were unaccounted for may have influenced the results. For example, we found differences between women in SHGs compared to women not in SHGs, with the former more marginalised. In addition, the uptake and adherence to the health layering component was not measured in this study, and should be documented in future work with SHGs. It may be that other factors that differentially influence more marginalised compared to less marginalised women, other than health promotion through SHGs, may have served to improve health indicators in women in SHGs. This requires evaluation in future studies. Future randomised, stepped-wedge or factorial designs could be used to examine health layering upon the government-led and scaled SHGs to further tease apart programmatic choices which would lead to greater efficiency of SHGs in promoting health, such as group size, facilitation including leader qualities, costs, and potential for sustainable impact [41]. We categorised SHG type according to the geolocation of the blocks, as we lacked data on whether groups belonged to a particular program (eg, *Parivartan* or JEEViKA). Further specification could elucidate whether groups for specific purposes such as agriculture or strictly microfinance were optimal for layering interventions. In addition, specific health layering modules could be developed and examined using randomisation in a stepped-wedge or adaptive trial design and measuring specific, program-targeted process measures, indicators and outcomes.

In summary, this study demonstrates the benefits of layering health interventions upon government-led SHGs at scale in Bihar, India. These results should be a call to action for the GoB and other state-led government agencies to capitalise on this platform for health change at scale.

## Additional material

Online Supplementary Document

## 

**Table:** **Figure 4**. Effect of SHG interventions on reproductive, maternal, newborn and child health, nutrition and sanitation indicators by continuum of care, CHS rounds 8-9 (2016-2017), comparing health-layered JEEViKA+HL SHG members vs non-members, 37 blocks (odds ratio, OR ± 95% confidence interval, CI) (left) and non-health-layered JEEViKA SHG members vs non-members, 433 blocks (OR ± 95% CI) (right). All models presented were adjusted for age of the mother and the sex of the focal child. These models also accounted for the study’s complex design by applying study weights. ANC - antenatal care, CHS – Community-based Household Survey, DPT – pertussis-tetanus, FLW – frontline worker, HL – health layering, IFA – iron-folic acid, IPV – inactivated polio vaccine, OPV – oral polio vaccine, PPIUD – postpartum intrauterine device, SHG – self-help group.
