# Supplementary material for: Health layering of self-help groups: impacts on reproductive, maternal, newborn and child health and nutrition in Bihar, India
Source: J Glob Health. 2020 Dec 19;10(2):021007. doi: 10.7189/jogh.10.021007 (PMC7759023; doi:10.7189/jogh.10.021007)
Supplement: Online Supplementary Document [file jogh-10-021007-s001.pdf]

Health layering of self-help groups: impacts on reproductive, maternal, newborn and child health and nutrition in Bihar, India

Supplemental Figure 1. Health Layering upon Self Help groups (SHGs) in Bihar, India 2014-2017

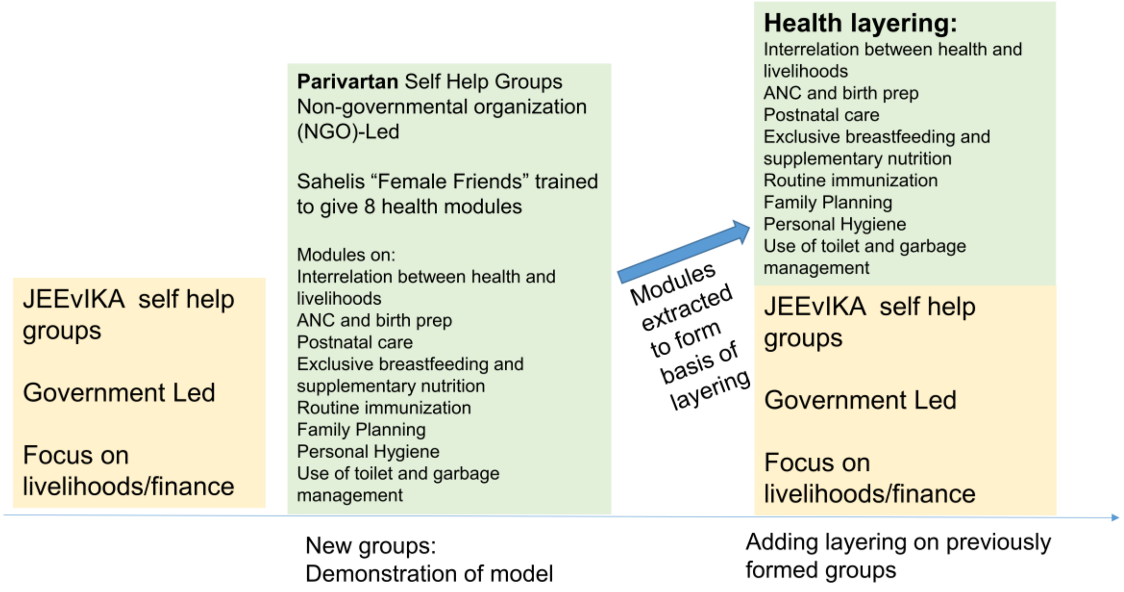

Supplemental Table 1a. Effect of SHG interventions on reproductive, maternal, newborn and child health and nutrition indicators by continuum of care, CHS round 6 (2014), comparing *Parivartan* SHG members vs non-members, 64 blocks (OR +/- 95% CI)									
				Round 6 (2014)					
**Description**	**Continuum of care domain**	**Delivery Platform**	**Effect**	**Odds Ratio Estimate**	**Lower 95% Confidence**	**Upper 95% Confidence**	**Higher odds for SHG in *Parivartan* blocks compared to non members**	**Equal odds for SHG in *Parivartan* blocks compared to non members**	**Lower odds for SHG in *Parivartan* blocks compared to non members**
4+ ANC visits	Antenatal Care	Facility/Outreach Service Delivery	Parivartan vs non SHG	0.786	0.425	1.456	0	1	0
Had at least one ANC exam if reporting any ANC visit	Antenatal Care	Facility/Outreach Service Delivery	Parivartan vs non SHG	2.104	0.289	15.29	0	1	0
Admitted to hospital for complication	Antenatal Care	Facility/Outreach Service Delivery	Parivartan vs non SHG	2.104	0.289	15.29	0	1	0
Received at least 90 IFA tablets during pregnancy	Antenatal Care	Facility/Outreach Service Delivery	Parivartan vs non SHG	1.723	1.183	2.508	1	0	0
FLW antenatal home visit to discuss mother's or baby's health	Antenatal Care	Frontline worker Performance	Parivartan vs non SHG	2.586	1.88	3.557	1	0	0
Any FLW visit during last trimester	Antenatal Care	Frontline worker Performance	Parivartan vs non SHG	2.567	1.87	3.524	1	0	0
FLW advised on hand-washing by delivery attendant	Antenatal Care	Frontline worker Performance	Parivartan vs non SHG	3.661	2.442	5.49	1	0	0
FLW adviced on danger of excessive bleeding	Antenatal Care	Frontline worker Performance	Parivartan vs non SHG	3.499	2.33	5.255	1	0	0
FLW advised on danger of convulsions	Antenatal Care	Frontline worker Performance	Parivartan vs non SHG	2.504	1.565	4.005	1	0	0
FLW advised on danger of prolonged or difficult labor	Antenatal Care	Frontline worker Performance	Parivartan vs non SHG	3.095	2.074	4.617	1	0	0
FLW advised on danger of swelling of face or hands	Antenatal Care	Frontline worker Performance	Parivartan vs non SHG	3.623	2.478	5.297	1	0	0
FLW advised on reasons to deliver in a hospital	Antenatal Care	Frontline worker Performance	Parivartan vs non SHG	1.444	0.84	2.48	0	1	0

FLW advised on saving money in case of emergency	Antenatal Care	Frontline worker Performance	Parivartan vs non SHG	2.366	1.632	3.43	1	0	0
Consumed 90+ IFA tablets	Antenatal Care	Mother's Behavior	Parivartan vs non SHG	1.187	0.739	1.905	0	1	0
Pregnancy registration in the first trimester	Antenatal Care	Mother's Behavior	Parivartan vs non SHG	2.018	1.481	2.749	1	0	0
Sought care for complications	Antenatal Care	Mother's Behavior	Parivartan vs non SHG	1.794	0.937	3.435	0	1	0
Saved money	Antenatal Care	Mother's Behavior	Parivartan vs non SHG	1.639	1.112	2.416	1	0	0
Chose a facility for delivery	Antenatal Care	Mother's Behavior	Parivartan vs non SHG	2.117	1.474	3.04	1	0	0
Chose a facility in case of emergency	Antenatal Care	Mother's Behavior	Parivartan vs non SHG	1.2	0.848	1.698	0	1	0
Arranged transportation to facility	Antenatal Care	Mother's Behavior	Parivartan vs non SHG	0.916	0.657	1.279	0	1	0
Delivery in a private facility (out of all deliveries)	Delivery	Facility/Outreach Service Delivery	Parivartan vs non SHG	0.66	0.377	1.154	0	1	0
Caesarian-section for delivery	Delivery	Facility/Outreach Service Delivery	Parivartan vs non SHG	0.709	0.327	1.54	0	1	0
Clean cloth was used for baby	Delivery	Facility/Outreach Service Delivery	Parivartan vs non SHG	1.807	0.545	5.988	0	1	0
Clean thread was used to tie cord	Delivery	Facility/Outreach Service Delivery	Parivartan vs non SHG	0.522	0.181	1.509	0	1	0
Baby immediately dried and wrapped	Delivery	Mother's Behavior	Parivartan vs non SHG	0.672	0.294	1.538	0	1	0
Any FLW visits in the first week after delivery	Postnatal Care	Frontline worker Performance	Parivartan vs non SHG	2.613	1.923	3.55	1	0	0
3+ FLW visits in the first week after delivery	Postnatal Care	Frontline worker Performance	Parivartan vs non SHG	2.619	1.87	3.666	1	0	0
FLW advised on neonatal danger signs	Postnatal Care	Frontline worker Performance	Parivartan vs non SHG	3.931	2.391	6.461	1	0	0
FLW advised on delayed bathing	Postnatal Care	Frontline worker Performance	Parivartan vs non SHG	3.798	2.602	5.543	1	0	0
FLW advised on skin to skin care	Postnatal Care	Frontline worker Performance	Parivartan vs non SHG	3.66	2.508	5.341	1	0	0

FLW advised on dry cord care	Postnatal Care	Frontline worker Performance	Parivartan vs non SHG	2.876	1.967	4.204	1	0	0
Skin-to-skin care	Postnatal Care	Mother's Behavior	Parivartan vs non SHG	3.229	2.335	4.465	1	0	0
Dry cord care	Postnatal Care	Mother's Behavior	Parivartan vs non SHG	2.819	2.028	3.919	1	0	0
Care seeking for neonatal complications	Postnatal Care	Mother's Behavior	Parivartan vs non SHG	0.815	0.28	2.376	0	1	0
FLW advised on early initiation of breastfeeding	Nutrition	Frontline worker Performance	Parivartan vs non SHG	2.979	1.915	4.634	1	0	0
FLW advised on exclusive breastfeeding	Nutrition	Frontline worker Performance	Parivartan vs non SHG	3.405	2.245	5.162	1	0	0
FLW advised on age to which to continuing breastfeeding	Nutrition	Frontline worker Performance	Parivartan vs non SHG	5.578	3.533	8.807	1	0	0
Immediate breastfeeding	Nutrition	Mother's Behavior	Parivartan vs non SHG	2.703	1.874	3.899	1	0	0
Exclusive breastfeeding in the past 24 hours	Nutrition	Mother's Behavior	Parivartan vs non SHG	2.604	1.711	3.963	1	0	0
FLW reminded on vaccine information	Immunization	Frontline worker Performance	Parivartan vs non SHG	1.797	1.078	2.995	1	0	0
FLW asked interest in having more children	Family Planning	Frontline worker Performance	Parivartan vs non SHG	2.273	1.493	3.461	1	0	0
FLW asked risk of becoming pregnant post-delivery	Family Planning	Frontline worker Performance	Parivartan vs non SHG	2.039	1.359	3.061	1	0	0
FLW advised on sterilization post-delivery	Family Planning	Frontline worker Performance	Parivartan vs non SHG	2.236	1.517	3.295	1	0	0
FLW advised on use of PPIUD post-delivery	Family Planning	Frontline worker Performance	Parivartan vs non SHG	2.662	1.765	4.016	1	0	0
Modern method of contraception used	Family Planning	Mother's Behavior	Parivartan vs non SHG	0.817	0.066	10.129	0	1	0
Washed hands before feeding child	Sanitation	Mother's Behavior	Parivartan vs non SHG	0.761	0.085	6.813	0	1	0
Used soap or detergent when washing hands before feed	Sanitation	Mother's Behavior	Parivartan vs non SHG	1.012	0.107	9.593	0	1	0

Supplemental Table 1a Legend:
All models presented were adjusted for age of the mother and the sex of the focal child. These models also accounted for the study's complex design by applying study weights. Abbreviations: ANC, antenatal care; CHS, Community-based Household Survey; CI Confidence Interval; DPT, diphtheria-pertussis-tetanus; FLW, frontline worker; HL, Health Layering; IFA, iron-folic acid; IPV, inactivated polio vaccine; OPV, oral polio vaccine; OR Odds Ratio; PPIUD, postpartum intrauterine device; SHG, self-help group

Supplemental Table 1b. Effect of SHG interventions on reproductive, maternal, newborn and child health and nutrition indicators by continuum of care, CHS rounds 8-9 (2016-2017), comparing SHG members vs non-members in 37 blocks with JEEViKA+HL layering (OR +/- 95% CI).									
				Round 8 and 9, 2015-2017					
**Description**	**Continuum of care domain**	**Delivery Platform**	**Effect**	**Odds Ratio Estimate**	**Lower 95% Confidence**	**Upper 95% Confidence**	**Higher odds for SHG in 37 JEEViKA + Health after to implementation compared to non members**	**Equal odds for SHG in 37 JEEViKA + Health after to implementation compared to non members**	**Lower odds for SHG in 37 JEEViKA + Health after implementation compared to non members**
4+ ANC visits	Antenatal Care	Facility/Outreach Service Delivery	JEEViKA+HL vs non SHGJEEViKA+HL vs non SHG	0.808	0.639	1.023	0	1	0
Had at least one ANC exam if reporting any ANC visit	Antenatal Care	Facility/Outreach Service Delivery	JEEViKA+HL vs non SHG	0.805	0.268	2.415	0	1	0
Admitted to hospital for complication	Antenatal Care	Facility/Outreach Service Delivery	JEEViKA+HL vs non SHG	0.805	0.268	2.415	0	1	0
Received at least 90 IFA tablets during pregnancy	Antenatal Care	Facility/Outreach Service Delivery	JEEViKA+HL vs non SHG	0.932	0.573	1.518	0	1	0
FLW antenatal home visit to discuss mother's or baby's health	Antenatal Care	Frontlineworker Performance	JEEViKA+HL vs non SHG	1.469	1.199	1.799	1	0	0
Any FLW visit during last trimester	Antenatal Care	Frontlineworker Performance	JEEViKA+HL vs non SHG	1.378	1.116	1.7	1	0	0
FLW advised on hand-washing by delivery attendant	Antenatal Care	Frontlineworker Performance	JEEViKA+HL vs non SHG	1.073	0.787	1.462	0	1	0
FLW adviced on danger of excessive bleeding	Antenatal Care	Frontlineworker Performance	JEEViKA+HL vs non SHG	0.977	0.666	1.433	0	1	0
FLW advised on danger of convulsions	Antenatal Care	Frontlineworker Performance	JEEViKA+HL vs non SHG	0.936	0.579	1.515	0	1	0
FLW advised on danger of prolonged or difficult labor	Antenatal Care	Frontlineworker Performance	JEEViKA+HL vs non SHG	1.049	0.694	1.586	0	1	0
FLW advised on danger of swelling of face or hands	Antenatal Care	Frontlineworker Performance	JEEViKA+HL vs non SHG	1.158	0.798	1.679	0	1	0
FLW advised on reasons to deliver in a hospital	Antenatal Care	Frontlineworker Performance	JEEViKA+HL vs non SHG	1.639	1.022	2.629	1	0	0
FLW advised on saving	Antenatal Care	Frontlineworker Performance	JEEViKA+HL vs non SHG	1.187	0.864	1.63	0	1	0

money in case of emergency									
Consumed 90+ IFA tablets	Antenatal Care	Mother's Behavior	JEEViKA+HL vs non SHG	0.854	0.474	1.538	0	1	0
Pregnancy registration in the first trimester	Antenatal Care	Mother's Behavior	JEEViKA+HL vs non SHG	1.193	0.971	1.464	0	1	0
Sought care for complications	Antenatal Care	Mother's Behavior	JEEViKA+HL vs non SHG	0.911	0.709	1.17	0	1	0
Saved money	Antenatal Care	Mother's Behavior	JEEViKA+HL vs non SHG	1.345	1.077	1.68	1	0	0
Chose a facility for delivery	Antenatal Care	Mother's Behavior	JEEViKA+HL vs non SHG	1.717	1.375	2.145	1	0	0
Chose a facility in case of emergency	Antenatal Care	Mother's Behavior	JEEViKA+HL vs non SHG	0.669	0.513	0.872	0	0	1
Arranged transportation to facility	Antenatal Care	Mother's Behavior	JEEViKA+HL vs non SHG	1.119	0.888	1.409	0	1	0
Delivery in a private facility (out of all deliveries)	Delivery	Facility/Outreach Service Delivery	JEEViKA+HL vs non SHG	0.778	0.587	1.032	0	1	0
Caesarian-section for delivery	Delivery	Facility/Outreach Service Delivery	JEEViKA+HL vs non SHG	0.821	0.547	1.232	0	1	0
Clean cloth was used for baby	Delivery	Facility/Outreach Service Delivery	JEEViKA+HL vs non SHG	1.186	0.448	3.136	0	1	0
Clean thread was used to tie cord	Delivery	Facility/Outreach Service Delivery	JEEViKA+HL vs non SHG	1.78	0.563	5.63	0	1	0
Baby immediately dried and wrapped	Delivery	Mother's Behavior	JEEViKA+HL vs non SHG	0.794	0.462	1.364	0	1	0
Any FLW visits in the first week after delivery	Postnatal Care	Frontlineworker Performance	JEEViKA+HL vs non SHG	1.29	1.052	1.582	1	0	0
3+ FLW visits in the first week after delivery	Postnatal Care	Frontlineworker Performance	JEEViKA+HL vs non SHG	1.152	0.849	1.564	0	1	0
FLW advised on neonatal danger signs	Postnatal Care	Frontlineworker Performance	JEEViKA+HL vs non SHG	0.586	0.224	1.535	0	1	0
FLW advised on delayed bathing	Postnatal Care	Frontlineworker Performance	JEEViKA+HL vs non SHG	1.19	0.876	1.617	0	1	0
FLW advised on skin to skin care	Postnatal Care	Frontlineworker Performance	JEEViKA+HL vs non SHG	1.304	0.94	1.81	0	1	0
FLW advised on dry cord care	Postnatal Care	Frontlineworker Performance	JEEViKA+HL vs non SHG	0.948	0.681	1.32	0	1	0
Skin-to-skin care	Postnatal Care	Mother's Behavior	JEEViKA+HL vs non SHG	1.183	0.908	1.54	0	1	0
Dry cord care	Postnatal Care	Mother's Behavior	JEEViKA+HL vs non SHG	1.072	0.872	1.319	0	1	0
Care seeking for neonatal complications	Postnatal Care	Mother's Behavior	JEEViKA+HL vs non SHG	3.515	1.584	7.8	1	0	0
FLW advised on early initiation of breastfeeding	Nutrition	Frontlineworker Performance	JEEViKA+HL vs non SHG				0	0	1

FLW advised on exclusive breastfeeding	Nutrition	Frontlineworker Performance	JEEViKA+HL vs non SHG	1.286	0.952	1.738	0	1	0
FLW advised on age to which to continuing breastfeeding	Nutrition	Frontlineworker Performance	JEEViKA+HL vs non SHG	1.199	0.881	1.631	0	1	0
Immediate breastfeeding	Nutrition	Mother's Behavior	JEEViKA+HL vs non SHG	1.569	1.243	1.981	1	0	0
Exclusive breastfeeding in the past 24 hours	Nutrition	Mother's Behavior	JEEViKA+HL vs non SHG	1.963	1.474	2.612	1	0	0
FLW reminded on vaccine information	Immunization	Frontlineworker Performance	JEEViKA+HL vs non SHG	1.182	0.933	1.497	0	1	0
FLW asked interest in having more children	Family Planning	Frontlineworker Performance	JEEViKA+HL vs non SHG	1.46	1.027	2.076	1	0	0
FLW asked risk of becoming pregnant post-delivery	Family Planning	Frontlineworker Performance	JEEViKA+HL vs non SHG	1.514	1.078	2.125	1	0	0
FLW advised on sterilization post-delivery	Family Planning	Frontlineworker Performance	JEEViKA+HL vs non SHG	1.441	1.041	1.994	1	0	0
FLW advised on use of PPIUD post-delivery	Family Planning	Frontlineworker Performance	JEEViKA+HL vs non SHG	1.032	0.703	1.515	0	1	0

Supplemental Table 1b Legend:
All models presented were adjusted for age of the mother and the sex of the focal child. These models also accounted for the study's complex design by applying study weights. Abbreviations: ANC, antenatal care; CHS, Community-based Household Survey; CI Confidence Interval; DPT, diphtheria-pertussis-tetanus; FLW, frontline worker; HL, Health Layering; IFA, iron-folic acid; IPV, inactivated polio vaccine; OPV, oral polio vaccine; OR Odds Ratio; PPIUD, postpartum intrauterine device; SHG, self-help group

Supplemental Table 1c. Effect of SHG interventions on reproductive, maternal, newborn and child health and nutrition indicators by continuum of care, CHS rounds 8-9 (2016-2017), comparing SHG members vs non-members in 433 *JEEViKA* blocks (OR +/- 95% CI).									
				Rounds 8 and 9, 2015-2017					
**Description**	**Continuum of Care Domain**	**Delivery Platform**	**Effect**	**Odds Ratio Estimate**	**Lower 95% Confidence**	**Upper 95% Confidence**	**Higher odds for SHG in JEEViKA blocks compared to non members**	**Equal odds for SHG in JEEViKA blocks compared to non members**	**Lower odds for SHG in JEEViKA blocks compared to non members**
4+ ANC visits	Antenatal Care	Facility/Outreach Service Delivery	JEEViKA ony vs non SHG	0.764	0.705	0.827	0	0	1
Had at least one ANC exam if reporting any ANC visit	Antenatal Care	Facility/Outreach Service Delivery	JEEViKA ony vs non SHG	0.577	0.347	0.959	0	0	1
Admitted to hospital for complication	Antenatal Care	Facility/Outreach Service Delivery	JEEViKA ony vs non SHG	0.577	0.347	0.959	0	0	1
Received at least 90 IFA tablets during pregnancy	Antenatal Care	Facility/Outreach Service Delivery	JEEViKA ony vs non SHG	1.202	1.038	1.391	1	0	0
FLW antenatal home visit to discuss mother's or baby's health	Antenatal Care	Frontlineworker Performance	JEEViKA ony vs non SHG	1.24	1.156	1.33	1	0	0
Any FLW visit during last trimester	Antenatal Care	Frontlineworker Performance	JEEViKA ony vs non SHG	1.254	1.167	1.348	1	0	0
FLW advised on hand-washing by delivery attendant	Antenatal Care	Frontlineworker Performance	JEEViKA ony vs non SHG	1.156	1.039	1.286	1	0	0
FLW adviced on danger of excessive bleeding	Antenatal Care	Frontlineworker Performance	JEEViKA ony vs non SHG	1.145	1.012	1.296	1	0	0
FLW advised on danger of convulsions	Antenatal Care	Frontlineworker Performance	JEEViKA ony vs non SHG	1.162	1.001	1.35	1	0	0
FLW advised on danger of prolonged or difficult labor	Antenatal Care	Frontlineworker Performance	JEEViKA ony vs non SHG	1.15	1.005	1.317	1	0	0
FLW advised on danger of swelling of face or hands	Antenatal Care	Frontlineworker Performance	JEEViKA ony vs non SHG	1.133	0.994	1.292	0	1	0
FLW advised on reasons	Antenatal Care	Frontlineworker Performance	JEEViKA ony vs non SHG	1.287	1.121	1.478	1	0	0

to deliver in a hospital									
FLW advised on saving money in case of emergency	Antenatal Care	Frontlineworker Performance	JEEViKA ony vs non SHG	1.121	1.004	1.251	1	0	0
Consumed 90+ IFA tablets	Antenatal Care	Mother's Behavior	JEEViKA ony vs non SHG	0.978	0.805	1.188	0	1	0
Pregnancy registration in the first trimester	Antenatal Care	Mother's Behavior	JEEViKA ony vs non SHG	1.109	1.033	1.191	1	0	0
Sought care for complication s	Antenatal Care	Mother's Behavior	JEEViKA ony vs non SHG	0.851	0.786	0.922	0	0	1
Saved money	Antenatal Care	Mother's Behavior	JEEViKA ony vs non SHG	1.122	1.037	1.214	1	0	0
Chose a facility for delivery	Antenatal Care	Mother's Behavior	JEEViKA ony vs non SHG	1.129	1.05	1.213	1	0	0
Chose a facility in case of emergency	Antenatal Care	Mother's Behavior	JEEViKA ony vs non SHG	0.931	0.852	1.017	0	1	0
Arranged transportatio n to facility	Antenatal Care	Mother's Behavior	JEEViKA ony vs non SHG	1.027	0.945	1.117	0	1	0
Delivery in a private facility (out of all deliveries)	Delivery	Facility/Outreac h Service Delivery	JEEViKA ony vs non SHG	0.589	0.53	0.654	0	0	1
Caesarian-section for delivery	Delivery	Facility/Outreac h Service Delivery	JEEViKA ony vs non SHG	0.673	0.578	0.785	0	0	1
Clean cloth was used for baby	Delivery	Facility/Outreac h Service Delivery	JEEViKA ony vs non SHG	1.078	0.798	1.456	0	1	0
Clean thread was used to tie cord	Delivery	Facility/Outreac h Service Delivery	JEEViKA ony vs non SHG	1.085	0.802	1.468	0	1	0
Baby immediately dried and wrapped	Delivery	Mother's Behavior	JEEViKA ony vs non SHG	1.145	0.922	1.421	0	1	0
Any FLW visits in the first week after delivery	Postnatal Care	Frontlineworker Performance	JEEViKA ony vs non SHG	1.201	1.119	1.289	1	0	0
3+ FLW visits in the first week after delivery	Postnatal Care	Frontlineworker Performance	JEEViKA ony vs non SHG	1.251	1.131	1.385	1	0	0
FLW advised on neonatal danger signs	Postnatal Care	Frontlineworker Performance	JEEViKA ony vs non SHG	1.231	0.996	1.521	0	1	0
FLW advised on delayed bathing	Postnatal Care	Frontlineworker Performance	JEEViKA ony vs non SHG	1.1	0.989	1.224	0	1	0
FLW advised on skin to skin care	Postnatal Care	Frontlineworker Performance	JEEViKA ony vs non SHG	1.092	0.975	1.224	0	1	0
FLW advised on dry cord care	Postnatal Care	Frontlineworker Performance	JEEViKA ony vs non SHG	1.142	1.022	1.277	1	0	0

Skin-to-skin care	Postnatal Care	Mother's Behavior	JEEViKA ony vs non SHG	1.211	1.109	1.323	1	0	0
Dry cord care	Postnatal Care	Mother's Behavior	JEEViKA ony vs non SHG	1.039	0.968	1.115	0	1	0
Care seeking for neonatal complications	Postnatal Care	Mother's Behavior	JEEViKA ony vs non SHG	1.066	0.902	1.258	0	1	0
FLW advised on early initiation of breastfeeding	Nutrition	Frontlineworker Performance	JEEViKA ony vs non SHG				0	0	1
FLW advised on exclusive breastfeeding	Nutrition	Frontlineworker Performance	JEEViKA ony vs non SHG	1.153	1.04	1.278	1	0	0
FLW advised on age to which to continuing breastfeeding	Nutrition	Frontlineworker Performance	JEEViKA ony vs non SHG	1.144	1.028	1.274	1	0	0
Immediate breastfeeding	Nutrition	Mother's Behavior	JEEViKA ony vs non SHG	1.304	1.205	1.41	1	0	0
Exclusive breastfeeding in the past 24 hours	Nutrition	Mother's Behavior	JEEViKA ony vs non SHG	1.083	0.997	1.177	0	1	0
FLW reminded on vaccine information	Immunization	Frontlineworker Performance	JEEViKA ony vs non SHG	1.059	0.976	1.149	0	1	0
Have immunisation card	Immunization	Facility/Outreach Service Delivery	JEEViKA ony vs non SHG				0	0	1
Polio (OPV3 or IPV) by card	Immunization	Facility/Outreach Service Delivery	JEEViKA ony vs non SHG				0	0	1
DPT3 by card	Immunization	Facility/Outreach Service Delivery	JEEViKA ony vs non SHG				0	0	1
FLW asked interest in having more children	Family Planning	Frontlineworker Performance	JEEViKA ony vs non SHG	1.261	1.104	1.441	1	0	0
FLW asked risk of becoming pregnant post-delivery	Family Planning	Frontlineworker Performance	JEEViKA ony vs non SHG	1.307	1.155	1.478	1	0	0
FLW advised on sterilization post-delivery	Family Planning	Frontlineworker Performance	JEEViKA ony vs non SHG	1.559	1.39	1.748	1	0	0
FLW advised on use of PPIUD post-delivery	Family Planning	Frontlineworker Performance	JEEViKA ony vs non SHG	1.291	1.133	1.472	1	0	0
Modern method of contraception used	Family Planning	Mother's Behavior	JEEViKA ony vs non SHG	1.339	1.231	1.457	1	0	0
Washed hands before feeding child	Sanitation	Mother's Behavior	JEEViKA ony vs non SHG	0.992	0.899	1.095	0	1	0

Washed hands after using toilet	Sanitation	Mother's Behavior	JEEViKA ony vs non SHG	1.64	0.977	2.754	0	1	0
Used soap or detergent when washing hands before feed	Sanitation	Mother's Behavior	JEEViKA ony vs non SHG	0.961	0.9	1.027	0	1	0
Used soap or detergent when washing hands after toilet	Sanitation	Mother's Behavior	JEEViKA ony vs non SHG	1.062	0.99	1.14	0	1	0

Supplemental Table 1c Legend:
All models presented were adjusted for age of the mother and the sex of the focal child. These models also accounted for the study's complex design by applying study weights. Abbreviations: ANC, antenatal care; CHS, Community-based Household Survey; CI Confidence Interval; DPT, diphtheria-pertussis-tetanus; FLW, frontline worker; HL, Health Layering; IFA, iron-folic acid; IPV, inactivated polio vaccine; OPV, oral polio vaccine; OR Odds Ratio; PPIUD, postpartum intrauterine device; SHG, self-help group

Supplemental Table 2. Comparison of reproductive, maternal, newborn and child health and nutritrion indicators for SHG members in 101 health-layered blocks compared to 433 JEEViKA 433 blocks, CHS rounds 8-9, 2015-2017									
**Description**	**Continuum of care domain**	**Delivery Platform**	**Effect**	**Odds Ratio Estimate**	**Lower 95% Confidence**	**Upper 95% Confidence**	**Higher odds for Health layering + JEEViKA compared to JEEViKA alone**	**Equal odds for Health layering + JEEViKA compared to JEEViKA alone**	**Lower odds for Health layering + JEEViKA compared to JEEViKA alone**
4+ ANC visits	Antenatal Care	Facility/Outreach Service Delivery	JEEViKA +HL vs SHG only	0.715	0.618	0.827	0	0	1
Had at least one ANC exam if reporting any ANC visit	Antenatal Care	Facility/Outreach Service Delivery	JEEViKA +HL vs SHG only	1.594	0.815	3.117	0	1	0
Admitted to hospital for complication	Antenatal Care	Facility/Outreach Service Delivery	JEEViKA +HL vs SHG only	1.594	0.815	3.117	0	1	0
Received at least 90 IFA tablets during pregnancy	Antenatal Care	Facility/Outreach Service Delivery	JEEViKA +HL vs SHG only	1.087	0.883	1.338	0	1	0
Delivery in a facility (public or private)	Delivery	Facility/Outreach Service Delivery	JEEViKA +HL vs SHG only	1.262	1.116	1.428	1	0	0
Delivery in a private facility (out of all deliveries)	Delivery	Facility/Outreach Service Delivery	JEEViKA +HL vs SHG only	0.926	0.776	1.106	0	1	0
Delivery in a public facility (out of all deliveries)	Delivery	Facility/Outreach Service Delivery	JEEViKA +HL vs SHG only	1.167	0.974	1.399	0	1	0
Caesarian-section for delivery	Delivery	Facility/Outreach Service Delivery	JEEViKA +HL vs SHG only	0.866	0.669	1.121	0	1	0
New blade was used to cut cord	Delivery	Facility/Outreach Service Delivery	JEEViKA +HL vs SHG only	0.849	0.306	2.359	0	1	0
Clean cloth was used for baby	Delivery	Facility/Outreach Service Delivery	JEEViKA +HL vs SHG only	1.404	0.914	2.157	0	1	0
Clean thread was used to tie cord	Delivery	Facility/Outreach Service Delivery	JEEViKA +HL vs SHG only	1.232	0.737	2.059	0	1	0
Baby weighed at birth	Delivery	Facility/Outreach Service Delivery	JEEViKA +HL vs SHG only	1.217	1.078	1.373	1	0	0
FLW antenatal home visit to discuss mother's or baby's health	Antenatal Care	Frontlineworker Performance	JEEViKA +HL vs SHG only	1.41	1.266	1.57	1	0	0
Any FLW visit during last trimester	Antenatal Care	Frontlineworker Performance	JEEViKA +HL vs SHG only	1.323	1.187	1.476	1	0	0

FLW advised on hand-washing by delivery attendant	Antenatal Care	Frontlineworker Performance	JEEViKA +HL vs SHG only	1.541	1.322	1.796	1	0	0
FLW adviced on danger of excessive bleeding	Antenatal Care	Frontlineworker Performance	JEEViKA +HL vs SHG only	1.102	0.913	1.33	0	1	0
FLW advised on danger of convulsions	Antenatal Care	Frontlineworker Performance	JEEViKA +HL vs SHG only	1.149	0.915	1.443	0	1	0
FLW advised on danger of prolonged or difficult labor	Antenatal Care	Frontlineworker Performance	JEEViKA +HL vs SHG only	1.308	1.072	1.596	1	0	0
FLW advised on danger of swelling of face or hands	Antenatal Care	Frontlineworker Performance	JEEViKA +HL vs SHG only	1.347	1.117	1.624	1	0	0
FLW advised on reasons to deliver in a hospital	Antenatal Care	Frontlineworker Performance	JEEViKA +HL vs SHG only	0.978	0.781	1.225	0	1	0
FLW advised on saving money in case of emergency	Antenatal Care	Frontlineworker Performance	JEEViKA +HL vs SHG only	1.232	1.049	1.446	1	0	0
Any FLW visits in the first week after delivery	Postnatal Care	Frontlineworker Performance	JEEViKA +HL vs SHG only	1.325	1.19	1.477	1	0	0
3+ FLW visits in the first week after delivery	Postnatal Care	Frontlineworker Performance	JEEViKA +HL vs SHG only	1.278	1.099	1.487	1	0	0
FLW advised on neonatal danger signs	Postnatal Care	Frontlineworker Performance	JEEViKA +HL vs SHG only	1.222	0.921	1.623	0	1	0
FLW advised on delayed bathing	Postnatal Care	Frontlineworker Performance	JEEViKA +HL vs SHG only	1.323	1.13	1.548	1	0	0
FLW advised on skin to skin care	Postnatal Care	Frontlineworker Performance	JEEViKA +HL vs SHG only	1.531	1.297	1.808	1	0	0
FLW advised on dry cord care	Postnatal Care	Frontlineworker Performance	JEEViKA +HL vs SHG only	1.56	1.329	1.832	1	0	0
FLW advised on early initiation of breastfeedin g	Nutrition	Frontlineworker Performance	JEEViKA +HL vs SHG only	1.799	1.227	2.637	1	0	0
FLW advised on exclusive breastfeedin g	Nutrition	Frontlineworker Performance	JEEViKA +HL vs SHG only	1.348	1.158	1.57	1	0	0
FLW advised on age to which to continuing breastfeedin g	Nutrition	Frontlineworker Performance	JEEViKA +HL vs SHG only	1.629	1.397	1.899	1	0	0
FLW reminded on vaccine information	Immuniz ation	Frontlineworker Performance	JEEViKA +HL vs SHG only	1.125	0.987	1.283	0	1	0

FLW asked interest in having more children	Family Planning	Frontlineworker Performance	JEEViKA +HL vs SHG only	1.252	1.04	1.506	1	0	0
FLW asked risk of becoming pregnant post-delivery	Family Planning	Frontlineworker Performance	JEEViKA +HL vs SHG only	1.245	1.044	1.485	1	0	0
FLW advised on sterilization post-delivery	Family Planning	Frontlineworker Performance	JEEViKA +HL vs SHG only	1.153	0.98	1.357	0	1	0
FLW advised on use of PPIUD post-delivery	Family Planning	Frontlineworker Performance	JEEViKA +HL vs SHG only	1.172	0.973	1.412	0	1	0
Consumed 90+ IFA tablets	Antenatal Care	Mother's Behavior	JEEViKA +HL vs SHG only	1.279	0.968	1.689	0	1	0
Pregnancy registration in the first trimester	Antenatal Care	Mother's Behavior	JEEViKA +HL vs SHG only	1.043	0.933	1.165	0	1	0
Sought care for complications	Antenatal Care	Mother's Behavior	JEEViKA +HL vs SHG only	1.131	0.992	1.288	0	1	0
Saved money	Antenatal Care	Mother's Behavior	JEEViKA +HL vs SHG only	1.162	1.028	1.313	1	0	0
Chose a facility for delivery	Antenatal Care	Mother's Behavior	JEEViKA +HL vs SHG only	1.26	1.122	1.415	1	0	0
Chose a facility in case of emergency	Antenatal Care	Mother's Behavior	JEEViKA +HL vs SHG only	1.17	1.026	1.334	1	0	0
Arranged transportation to facility	Antenatal Care	Mother's Behavior	JEEViKA +HL vs SHG only	1.308	1.158	1.478	1	0	0
Baby immediately dried and wrapped	Delivery	Mother's Behavior	JEEViKA +HL vs SHG only	0.93	0.678	1.276	0	1	0
Skin-to-skin care	Postnatal Care	Mother's Behavior	JEEViKA +HL vs SHG only	1.28	1.118	1.465	1	0	0
Dry cord care	Postnatal Care	Mother's Behavior	JEEViKA +HL vs SHG only	1.358	1.217	1.516	1	0	0
Care seeking for neonatal complications	Postnatal Care	Mother's Behavior	JEEViKA +HL vs SHG only	1.599	1.161	2.203	1	0	0
Immediate breastfeeding	Nutrition	Mother's Behavior	JEEViKA +HL vs SHG only	1.338	1.185	1.51	1	0	0
Exclusive breastfeeding in the past 24 hours	Nutrition	Mother's Behavior	JEEViKA +HL vs SHG only	1.379	1.203	1.58	1	0	0
Initiation of complementary feeding	Nutrition	Mother's Behavior	JEEViKA +HL vs SHG only	0.613	0.252	1.491	0	1	0
Age-appropriate initiation of complementary feeding	Nutrition	Mother's Behavior	JEEViKA +HL vs SHG only	2.369	0.306	18.332	0	1	0

(6-8 months of age)									
Age-appropriate frequency of complementary feeding (3+ times for 9-11 month-old children)	Nutrition	Mother's Behavior	JEEViKA +HL vs SHG only	0.724	0.26	2.017	0	1	0
Fed complementary cereal-based food in past 24 hours	Nutrition	Mother's Behavior	JEEViKA +HL vs SHG only	0.609	0.25	1.481	0	1	0
Modern method of contraception used	Family Planning	Mother's Behavior	JEEViKA +HL vs SHG only	1.848	0.687	4.97	0	1	0
Modern method of contraception used	Family Planning	Mother's Behavior	JEEViKA +HL vs SHG only	1.848	0.687	4.97	0	1	0
Washed hands before feeding child	Sanitation	Mother's Behavior	JEEViKA +HL vs SHG only	1.407	0.327	6.062	0	1	0
Used soap or detergent when washing hands before feed	Sanitation	Mother's Behavior	JEEViKA +HL vs SHG only	0.659	0.267	1.631	0	1	0
Used soap or detergent when washing hands after toilet	Sanitation	Mother's Behavior	JEEViKA +HL vs SHG only	0.249	0.056	1.104	0	1	0

Supplemental Table 2 Legend:
All models presented were adjusted for age of the mother and the sex of the focal child. These models also accounted for the study’s complex design by applying study weights. Abbreviations: ANC, antenatal care; CHS, Community-based Household Survey; CI Confidence Interval; DPT, diphtheria-pertussis-tetanus; FLW, frontline worker; HL, Health Layering; IFA, iron-folic acid; IPV, inactivated polio vaccine; OPV, oral polio vaccine; OR Odds Ratio; PPIUD, postpartum intrauterine device; SHG, self-help group
JEEViKA+HL vs SHG only *is an indicator which compares SHG members in the 101 blocks with Health Layering either Parivartan or JEEViKA + HL to SHG members in the 433 blocks with JEEViKA alone, Round 8 and 9, 2015-2017 only*

Supplemental Table 3. Timeline of Interventions and Evaluations				
**Time- Evaluation Rounds**	**64 blocks- Parivartan**	**37 blocks JEEViKA+ Health (101 total) (round 6 only, control 2)**	**256 Light touch blocks**	**433 blocks, rest of bihar (control 1)**
Round 6- May 2104- Sept 2014	Active	JEEViKA	JEEViKA	JEEViKA
Round 7 Sept 2015- December 2015	Active	Transition	Transition	JEEViKA
Round 8 October 2015- Jan 2016	Active	Active	JEEViKA or Active?	JEEViKA
Round 9 October 2016- Jan 2017	Phase out- transition to JEEViKA+ Health	Active	Active	JEEViKA

Comparison 1: SHG to non SHG in 64 Parivartan blocks (Rounds 6- 9 together)
Comparison 2: SHG to non SHG in 37 JEEViKA + Health blocks (Rounds 6- 9 together)
Comparison 3: SHG to non SHG in 433 JEEViKA blocks (Rounds 6- 9 together)
Comparison 4: SHG members in 101 Health-layered blocks (Parivartan and JEEViKA+HL) compared to SHG members in JEEViKA in 433 blocks in the rest of Bihar
SHG=Self Help Group
